# Patterns and Consequences of Male–Infant Relationships in Wild Assamese Macaques (*Macaca assamensis*)

**DOI:** 10.1007/s10764-016-9904-2

**Published:** 2016-05-06

**Authors:** Christin Minge, Andreas Berghänel, Oliver Schülke, Julia Ostner

**Affiliations:** 1 Department for Behavioral Ecology, Georg-August University Göttingen, 37077 Göttingen, Germany; 2 Research Group Primate Social Evolution, German Primate Center Göttingen, 37077 Göttingen, Germany

**Keywords:** Anti-harassment hypothesis, Anti-infanticide hypothesis, Assamese macaques, Long-term male–immature association, Mating effort, Paternal care

## Abstract

Male care for offspring is unexpected in polygynandrous mammals. Evidence from nonhuman primates, however, indicates not only the existence of stable male–immature associations in multimale–multifemale groups, but also male care in the form of protection from infanticidal attacks and conspecific harassment. Here, we investigate the relationship characteristics, dynamics, and consequences of male–immature associations in wild Assamese macaques, *Macaca assamensis*, at Phu Khieo Wildlife Sanctuary, Thailand, to inform hypotheses of their evolutionary origins. Female Assamese macaques reproduce seasonally and do not signal ovulation, resulting in low mating and paternity skew. However, male–immature associations are predicted by paternity, and male behavior potentially reflects paternal effort. We present focal animal data on 12 immatures followed from birth beyond weaning into their juvenile life (1188 focal hours). The distribution of composite sociality indices suggests that male–immature relationships were highly differentiated. Association patterns and the degree of differentiation remained stable from 6 mo well into the juvenile phase, suggesting that male protection extends beyond the phase of high infanticide risk. Based on Hinde indices, immatures were responsible for maintaining the relationships. The likelihood that an infant was associated with its preferred male increased if the mother was absent and if other males were present in proximity, suggesting that immatures sought protection. The presence of the preferred male did not decrease the rate of mild aggression immatures received from group members, but the stronger the relationship between an immature and a male, the more often it received agonistic support from him. Future studies will have to assess whether this agonistic support translates into improved fitness and represents true paternal care.

## Introduction

Most mammalian males do not provide care for infants and —if present at all after conception— associate and interact with immatures only rarely (van Schaik and Kappeler 1997). The rarity of male care for offspring has been explained by the high costs of missed mating opportunities and by a high degree of paternity uncertainty in polygynandrous mating systems (Clutton-Brock and Parker 1992; Trivers 1972). Yet, in primates, especially in species of the cercopithecine subfamily, in which males and females are associated year-round and typically live in polygynandrous multimale–multifemale groups (Cords 2012), males and immatures are frequently found in close spatial association and affiliative or supportive interaction (Maestripieri 1998). Variation within and between cercopithecine species in the degree of immature–male association and its consequences has not been fully explored yet.

When investigating evolutionary explanations for male–immature associations it has proven useful to separate male reproductive investments into mating effort and paternal effort (Muller and Emery Thompson 2012). The paternal care hypothesis proposes that associations reflect care provided by the male for his current offspring, thereby increasing the offspring’s chances of survival and consequently the provider’s fitness (Trivers 1972). Instead of directly caring for infants, e.g., carrying and food sharing, males may protect their offspring from conspecific threats to the physical integrity and survival of offspring (Buchan *et al*. 2003; van Schaik and Paul 1996). Conspecific threats may become manifest in increased injury risk from harassment by other group members (Altmann 1980; de Ruiter *et al*. 1994; Kleindorfer and Wasser 2004; Shopland and Altmann 1987; Smuts 1985; van Noordwijk and van Schaik 1988) as well as infanticide. Like other mammals in which time spent lactating exceeds the time spent gestating, primate infants face a high risk of infanticide by males (van Noordwijk and van Schaik 2000; van Schaik 2000a). High skew in mating and paternity success further increases the risk of infanticide (Henzi and Barrett 2003; Ostner *et al*. 2013; Palombit 2000, 2003).

Studies of primates living in multimale–multifemale groups provide mixed evidence for the paternal care hypothesis. For true paternal care to evolve males need to accurately distinguish their offspring from other infants (Alberts and Fitzpatrick 2012). In polygynandrous species males may assess paternity probability based on their own mating behavior, association history with the infant’s mother, and possibly phenotype matching (Borries *et al*. 1999; Buchan *et al*. 2003; Busse and Hamilton 1981; Charpentier *et al*. 2008; Lemasson *et al*. 2008; Moscovice *et al*. 2009, 2010; Ostner *et al*. 2013; Palombit *et al*. 1997; van Schaik 2000b; Widdig 2007). Playback experiments with chacma baboons (*Papio ursinus*) suggest that males respond more strongly to playbacks of a mother’s distress calls if they are in a close affiliative relationship with the female (Palombit *et al*. 1997). Together with the fact that chacma baboon females form close affiliative relationships or “friendships” with sires and likely sires of current offspring (Moscovice *et al*. 2009) and that these relationships and the associated support by the male “friend” end abruptly on the death of the offspring (Palombit *et al*. 1997), these experiments suggest that male behavior evolved as paternal protection against conspecific threat.

Chacma baboon males preferentially support both related and those unrelated juveniles with whose mother they previously had formed a close affiliative relationship (Moscovice *et al*. 2009) and provide enhanced access to food and undisturbed feeding (Huchard *et al*. 2013). Yellow baboon males (*Papio cynocephalus*) also preferentially support their genetic offspring in agonistic conflicts during the juvenile phase (Buchan *et al*. 2003) and the presence of the sire accelerates offspring maturation (Charpentier *et al*. 2008), which may result from accelerated socialization or reduced social stress (Fairbanks 1993). In rhesus (*Macaca mulatta*) and Assamese macaques (*Macaca assamensis*) sires associate more with their dependent genetic offspring than with other infants (Langos *et al*. 2013; Ostner *et al*. 2013), but rhesus macaque sires fail to support their genetic offspring in conflicts against other group members (Kulik *et al*. 2012). Male–immature associations are not predicted by paternity in Barbary macaques (*Macaca sylvanus*: Ménard *et al*. 2001; Paul *et al*. 1996) and mountain gorillas (*Gorilla beringei*: Rosenbaum *et al*. 2015).

The mating effort hypothesis for the evolution of male care proposes that male–immature associations evolved as a form of male mating effort (Seyfarth 1978) rather than paternal effort, with the male endearing himself to the female to enhance his future mating success with her. Accordingly, the hypothesis predicts that male–infant associations and male care will evolve independently of paternity as a mere by-product of male mating effort (Ménard *et al*. 2001; Ostner *et al*. 2013; Smuts 1985; van Schaik and Paul 1996). The mating effort hypothesis is supported by long-term studies of wild Assamese macaques, free-ranging and captive rhesus macaques, and wild chimpanzees (*Pan troglodytes*), in which male–infant or male–mother association predicted male mating success (Kulik *et al*. 2012; Langos *et al*. 2013; Massen and Sterck 2013; Massen *et al*. 2012; Ostner *et al*. 2013; Smuts 1985). In all of these species it has recently been shown that male*–*female relationships are stable well beyond one female reproductive cycle (Haunhorst *et al*. 2016; Langergraber *et al*. 2013; Massen and Sterck 2013; Ostner *et al*. 2013). A close heterosexual relationship may be formed at some point, increase current and/or future mating success, and remain stable through the gestation period and on into early infancy of the subsequent offspring, which often but not always is sired by the male “friend” (Ostner *et al*. 2013). Thus male–female association in these species could potentially reflect both mating effort and paternal effort. To date, most studies of male care focused either only on the first few weeks or months of an infant’s life (Huchard *et al*. 2010; Lemasson *et al*. 2008; Nguyen *et al*. 2009) or on the juvenile life phase alone (Buchan *et al*. 2003; Charpentier *et al*. 2008; Huchard *et al*. 2013; Moscovice *et al*. 2009), and many studies lack details on the nature of male–immature social relationships.

In Assamese macaques patterns of male–female and male–immature spatial association are consistent with the paternal care hypothesis as well as a modified version of the mating effort hypothesis, the “friends-with-benefits” hypothesis, which proposes that male–female relationships evolve for male mating benefits with the female “friend” without assuming that the relationship necessarily has to form prior to the mating season (Ostner *et al*. 2013). The different evolutionary routes to male–immature association produce different relationship characteristics, i.e., differences concerning the responsibility for relationship maintenance and relationship stability. In Assamese macaques we currently lack an understanding of the quality of male–immature relationships beyond spatial patterns as well as the possible benefits accruing for infants from selectively associating with adult males.

Reproduction in Assamese macaques is seasonal and females exhibit approximately 1- or 2-yr interbirth intervals (Fürtbauer *et al*. 2011a; Ostner *et al*. 2013). Male mating skew and paternity skew are low (Ostner *et al*. 2011; Sukmak *et al*. 2014). Infanticide has been directly observed (Kalbitz, Ostner, Schülke *unpubl. data*) and could be adaptive because male tenures are long (Ostner *et al*. 2013) and the chance for a female to conceive in the same year increases from 38% if the infant survives to 75% if the infant is lost before weaning (Schülke and Ostner *unpubl. data*). The risk of infanticide from within the group is reduced, however, because ovulation is concealed from males (Fürtbauer *et al*. 2011a) and females exhibit high mating synchrony (Fürtbauer *et al*. 2011b) and mate with all males in the group, leaving each of them with a nonzero chance of paternity for every infant. Although the timing of ovulation is concealed from males, males seem to infer some paternity certainty based on past mating history with the infant’s mother (Ostner *et al*. 2013).

Here we analyze immature focal animal data collected over the first 21 mo of an immature’s life and determine the distribution of the strength of male–immature affiliative relationships (see Table I for a summary of hypotheses and predictions). Based on our previous finding of selective male–immature associations (Ostner *et al*. 2013) we expect affiliative relationships to be differentiated, i.e., stronger and more consistent relationships in a few male–immature dyads. If *male care evolved as mating effort* alone, we predict that 1) males are mainly responsible for maintaining close proximity (<1.5 m) to the infant and 2) males should terminate their relationship with the infant as soon as the infant’s mother conceives again. If *male care evolved as paternal effort* alone the time spent in association may vary with the risks the offspring faces, but the degree of preference for their preferred offspring relative to other infants should remain constant. Specifically, if *male care serves an anti-infanticide function* we predict 1) that differentiation in male–immature social relationships is stronger during the preweaning (mo 1–12) compared to the postweaning period (mo 13–21) and peaks during the first 6 mo of life when the risk of infanticide is highest; 2) that the total time spent in proximity (≤5 m) to the preferred male is highest during the first 6 mo of life and decreases with increasing age/decreasing infanticide risk; and 3) that the presence of the preferred male in proximity of ≤5 m in the preweaning period (mo 1–12) is negatively associated with the presence of the mother and positively with the presence of nonpreferred males, because infanticide risk is higher in the absence of the mother and around nonpreferred males. If *male care serves an anti-harassment function*, we predict 1) that the total time spent in proximity to the preferred male remains constant and association strength remains high across the entire study period, as immatures, owing to their slow development, are still vulnerable to threats from aggressive conspecifics even after reaching independence; 2) that the presence of the preferred male in proximity of ≤5 m in both the pre- and the postweaning period is predicted negatively by the presence of the mother and positively by the presence of nonpreferred males, because the risk of harassment by aggressive conspecifics increases in the absence of the mother and the presence of an nonpreferred male. Most importantly, we predict 3) that the presence of the preferred males in proximity (≤5 m) decreases immatures’ rate of aggression received from and submission given to group members and 4) that preferred males support immatures in conflicts more often than nonpreferred males do.

**Table I:** Summary of hypotheses and predictions

Hypothesis	Prediction	Supported?
Mating effort	1) Males are mainly responsible for maintaining close proximity (≤1.5 m) to infant.	No
	2) The male–immature relationship ends with the mother’s conception.	No
Paternal effort	The degree of preference for preferred offspring relative to other infants remains constant.	Yes
Against infanticide	1) Differentiation in male–immature relationships is stronger during preweaning compared to postweaning period and peaks during first 6 mo of life.	Yes / no
	2) Total time spent in proximity with the PM is highest during the first 6 mo of life and decreases with increasing age.	No
	3) The presence of the PM in proximity in the preweaning period is negatively associated with the presence of the mother and positively with the presence of nonpreferred males.	Yes
Against harrassment	1) Total time spent in proximity to the PM is constant and association strength remains high across the entire study period.	Yes
	2) The presence of the PM in proximity in the pre- and postweaning period is predicted negatively by the presence of the mother and positively by the presence of nonpreferred males.	Yes
	3) The presence of the PM in proximity decreases immatures’ rate of aggression received from and submission given to group members.	No
	4) PMs support immatures in conflicts more often than nonpreferred males do.	Yes

See text for further explanation.

PM = preferred male.

## Materials and Methods

### Study Site and Study Group

We conducted the study at Phu Khieo Wildlife Sanctuary (PKWS, 16°05–35′N and 101°20–55′E) in northeastern Thailand, an area of maximal protection status that is part of the contiguous *ca.* 6500 km^2^ protected Western Isaan Forest Complex (Koenig *et al*. 2004). The forest at the study site Huai Mai Sot Yai (16°27′N, 101°38′E, 600–800 m a.s.l.) comprises mainly hill and evergreen forest with dry dipterocarp patches and bamboo stands (Borries *et al*. 2002). Its vegetation is dense and the terrain hilly (Schülke *et al*. 2011). Large populations of large herbivores such as elephants and gaurs as well as a diverse community of predators suggest the habitat is relatively intact (Kumsuk *et al*. 1999). Assamese macaques feed mainly on fruit from a large number of tree species and spend around 60% of their active time in the middle and upper strata of the forest (Heesen *et al*. 2013; Schülke *et al*. 2011).

We collected observational data on 12 immatures (all born in 2011; see Table II) from May 2011 through December 2012 from a fully habituated group of wild Assamese macaques that comprised 60 individuals in the 2011 birth season (9 adult males, 15 adult females, 1 subadult male, 23 juveniles, 12 infants) and 65 individuals (10 adult males, 15 adult females, 40 immatures) in 2012 (Berghänel *et al*. 2015). We classified immatures as infants until 12 mo of age, and as juveniles from 13 mo onwards. Infants included here were born during the study (8 cases) or 1–2 mo prior (4 cases). Date of birth was known from demographic monitoring of the group (10 cases exact day, 2 cases estimated as the midpoint of a 4- to 9-day period; Table II). The study group split in late August 2012 when 11 individuals (3 adult males, 4 adult females with their offspring born in 2011) emigrated from the main group (Table II).

**Table II:** Twelve focal infants, birth dates, focal time, and group residency of Assamese macaques at Phu Khieo Wildlife Sanctuary

Sex	Exact date of birth	Focal hours	Left group August 2012
Female	March 23, 2011	95.8	N
Male	April 10, 2011	101.5	Y
Female	April 20, 2011	92.1	Y
Female	April 20, 2011	103.5	N
Male	April 25, 2011	103.3	Y
Male	May 12, 2011^a^	106.8	N
Female	May 15, 2011^b^	100.6	N
Male	May 29, 2011	95.9	Y
Male	June 9, 2011	101.5	N
Female	June 11, 2011	93.1	N
Female	June 16, 2011	98.5	N
Male	July 2, 2011	95.8	N

^a^Midpoint between May 9 and May 16.

^b^Midpoint between May 14 and May 17.

### Behavioral Data Collection

Throughout the 20-mo study period we collected behavioral data almost daily using 30-min focal animal sampling (Altmann 1974). We followed all 12 immatures born in 2011 during all-day follows of the group from sleeping tree to sleeping tree (range: 92.1–106.8 h focal animal sampling per individual, total = 1188h). We continuously recorded all social behaviors (agonistic and affiliative) to measure frequencies and durations. At 10-min intervals we recorded the identities of all group members within 5 m of the focal individual, i.e., 5 m proximity. For the analysis of differentiation and relationship maintenance (Composite Sociality and Hinde indices) we included only social interactions that derived from individual approaches/departures (within 1.5 m, i.e., close proximity) of the immatures themselves to/from males and vice versa in our analysis; i.e., we excluded interactions wherein immatures were carried by conspecifics into proximity or into interactions with others to restrict our analysis to purely infant-related interactions. Sample size for the analyses varied depending on availability of the respective type of data.

### Differentiation of Immature–Male Relationships

**Fig. 1:**
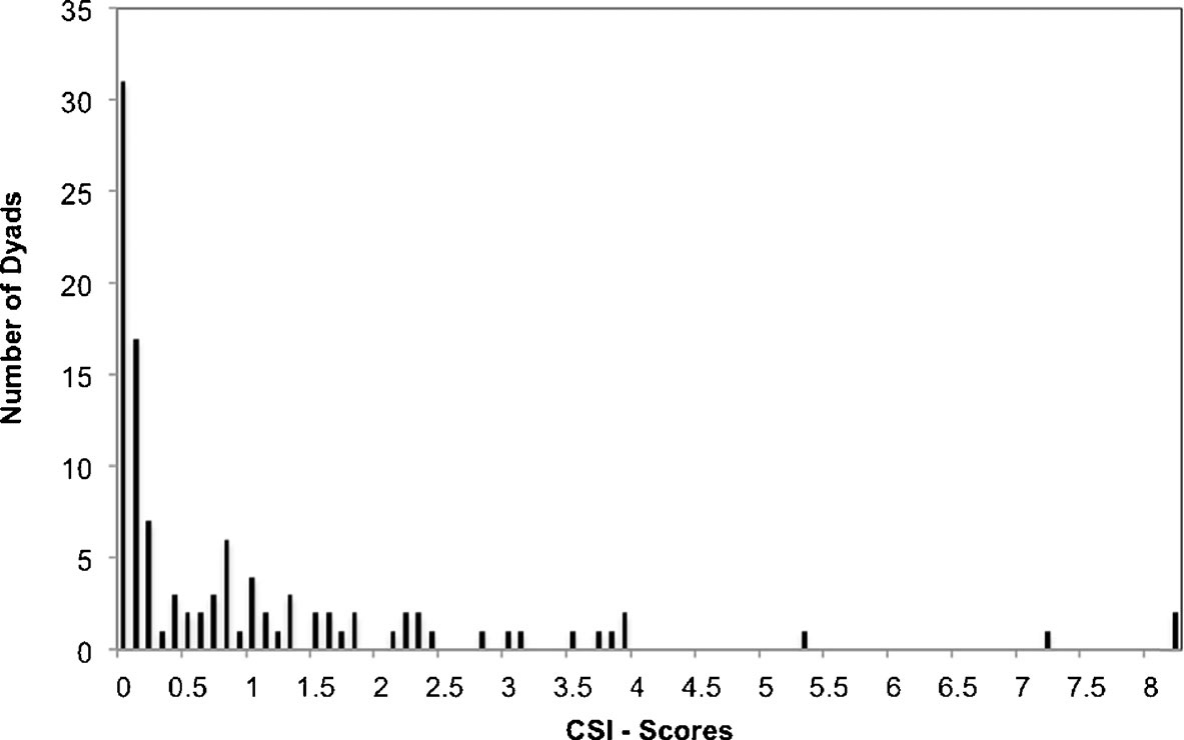
Differentiation of immature–male social relationships in a histogram of CSI scores of all immature–male dyads (*N* = 108) of one group of Assamese macaques at Phu Khieo Wildlife Sanctuary as a measure of the strength of an affiliative relationship (data from May 2011 to December 2012). The median is 0.2, 10% of scores exceeded 3.0, and the mean is by definition 1.

We calculated the Composite Sociality Index (CSI; Silk *et al*. 2006) for each adult male–immature dyad based on continuous focal sampling records from the entire study period to determine the strength of the association between each male and immature. Because the study group split in August 2012 we corrected male–immature dyadic focal time for the time the dyad coresided in the same group. Our CSI describes the relative strength of an affiliative relationship based on the frequency (*f*) and duration (*d*) of close spatial proximity of ≤1.5 m (*P*) and body contact (*B*) for each male–immature dyad relative to the mean across all dyads (CSI = (*Pd*_ij_*/Pd*_mean_ + *Pf*_ij_*/Pf*_mean_ + *Bd*_ij_*/Bd*_mean_ + *Bf*_ij_*/Bf*_mean_)/4). Grooming was too rare to be included, i.e., the mean number of grooming interactions per dyad was close to 1, yielding an unduly high influence of a single observed event on the resulting CSI value. All components included in the CSI were correlated in row-wise matrix correlations with 10,000 permutations using Kendall-τ correlations (mean ρ_rw,ave_ = 0.63 ± 0.18; range ρ_rw,ave_ = 0.49–0.87). All values were corrected for observation time and we subtracted approaches followed by body contact from the first and second term. Dyads with values >1 had a stronger relationship than the average male–immature dyad in the group (Fig. 1). The male with the highest CSI score, i.e., the top partner, was classified as an immature’s preferred male partner. For 11 of the 12 infants the preferred male partner had an above-average relationship with the infant, i.e., a CSI >1 (see Results).

### Responsibility for Maintaining Immature–Male Relationship

For every immature–male dyad with >20 tolerated approaches, i.e., approaches not leading to an agonistic interaction (Moscovice et al. 2009; Palombit *et al*. 1997), we calculated the Hinde Index (Hinde and Atkinson 1970) to determine which partner was responsible for the maintenance of close spatial association (≤1.5 m). Our Hinde Index is based on the number of approaches (*A*) by the immature (i) or the male (m) as well as the number of departures (*D*) by either individual (Hinde Index = (*A*_i_/(*A*_i_ + *A*_m_) – (*D*_i_/(*D*_i_ + *D*_m_)) × 100). Values of the Hinde Index range from –100 to +100. Positive values (above +10) indicate responsibility of the immature and high negative values (below – 10) responsibility of the male (Hill 1987). The number of dyads with >20 tolerated approaches was 40 across the entire study period. We also compared mean Hinde indices of the respective preferred male–immature dyads with the means across all other possible male–immature dyads in the group.

### Temporal Stability of Differentiation and Partner Choice in Immature–Male Associations

To assess the temporal stability of immatures’ choice of preferred partners we calculated the Consistency Index, modified from Silk *et al*. (2010) as follows: *C* = (*Y* – *U*)/(*Y* – 1) with *Y* = number of months that the immature was present and *U* = the number of different males that were an immature’s preferred male across different months of life. The values of C range from 0 (different partners in each month) to 1 (same partner across all months of life).

To assess the temporal stability of the differentiation in immature–male associations we split the data into month of life blocks and calculated an Association Index for each immature that expressed how often and how long it spent time in close proximity to its preferred male relative to the averages across other males in that month of an immature’s life. Specifically, for each month and immature we divided the frequency of time spent within 1.5 m of the preferred male (controlled for focal observation time) by the average frequency of time spent within 1.5 m of any other male. The same was done for duration of time spent within 1.5 m; the two terms were then summed and divided by 2 (*AI* = (*Pd*_ij_/*Pd*_mean_ + *Pf*_ij_/*Pf*_mean_)/2). The Association Index could not be calculated for some months for some immatures, because the average frequency of association was close to 1, rendering the measure inaccurate. In our comparison of relationship differentiation across month of life we included months with values for at least 7 immatures, i.e., excluded months 1, 2, 20, and 21 from this analysis (mean number of immatures per month ± SD: 10.52 ± 1.42). Thus, we report mean Association Indices with preferred males for the periods from 3 to 6 mo (highest infanticide risk), 7–12 mo (moderate infanticide risk), and 13–19 mo of immature age (period after weaning).

### Influence of Preferred Male’s Presence on Immature Aggression Received (and Submission Given)

To investigate whether the presence of the preferred male affected the rate of aggression immatures received, we created a dataset of 887 pairs of observations around proximity scans that differed only by the presence/absence of the preferred male and were otherwise matched for the age–sex composition of all individuals within 5 m of the immature focal individual, i.e., the pairs of observations were matched for the number of adult males, adult females, juveniles, and infants as well as mother present/absent. We then tested whether an immature received aggression or in a second model gave submission in an observation depending on whether or not the preferred male was around at this point in time.

### Male Agonistic Support of Immatures

We investigated the relationship between the strength of male–immature relationships based on the CSI and the frequency of support (counts) the infant received from the male in conflicts with other group members.

### Statistical Analysis

All statistical analyses were performed using R.2.1.4 (R development core team 2011). We used generalized linear mixed models (GLMMs; Baayen 2008) to investigate whether the presence of a second male (potential aggressor) and the mother (potential protector) in proximity (≤5 m) predicted the presence/absence of the preferred male in the proximity of the immature, with the presence of a second male as well as the mother (binomial) as fixed effects. Immature focal identity (to account for nonindependence of repeated observations within individuals), immature sex, and age–sex composition of group of other individuals within 5 m proximity of the focal immature were set as random factors. We ran the GLMM three times for different time periods of an immature’s life (entire study period: 1–21 mo; preweaning: 1–12 mo; postweaning: 13–21 mo).

To analyze whether the presence of the preferred male predicted the occurrence of aggression received or the rate of submission given by the immature (inclusive and exclusive of the aggression given by/submission given toward the preferred male), we ran GLMMs with the presence of the preferred male (binomial) as the fixed effect and immature identity, immature sex, and age–sex composition of the group of other individuals within 5 m proximity of the focal immature as random factors. GLMMs were run using the function lmer from the R package lme4 (Bates *et al*. 2011). GLMMs were fitted with binomial error structure and logit link function. Using a likelihood test (R function anova, package stats), we determined the statistical significance of the full model by comparing its fit with that of a null model including only the intercept and the random effects. Additional analyses revealed no strong correlation among predictors (Pearson correlation) and no violation of model assumption due to overdispersion, and influential cases using functions dfbeta and dffits.

Finally, we ran a GLMM with the number of times a male supported an immature in agonistic conflicts as the response, immature identity, and male identity as random factors and the *z*-transformed CSI value of the immature–male dyad as a fixed effect. We followed the same procedures mentioned previously but fitted a Poisson model. For all comparisons between different life phases we used two-sided Wilcoxon’s signed rank tests. Owing to small sample sizes we used exact tests as recommended by Mundry and Fischer (1998).

## Ethical Note

This study was undertaken completely noninvasively and with permission from the Department of National Parks, Wildlife and Plant Conservation (DNP) and the National Research Council of Thailand (NRCT) (permit: 2008/045).

## Results

### Characterization of Male–Immature Relationships

Across the 108 male–immature dyads relationships were clearly differentiated as indicated by a strongly skewed distribution of CSI scores, with a median of 0.2, well below the mean of 1 and 10% of the dyads featuring scores above 3 (Fig. 1). Across infants the mean CSI of the top partner, the single preferred male, was 3.5, >2 standard deviations from the mean of 1. The CSI scores for one immature were all <1 and we chose the male with the highest CSI (CSI = 0.8) as preferred male. For six infants the CSI scores with the top two males differed by <0.5. For our analyses we defined the male with the higher CSI as the single preferred male; however, we run all analyses again with these runner-up males, i.e., the second top males (separated by <0.5 in CSI score from top preferred), which did not significantly change our results or conclusions. For the other six immatures the CSI values for the preferred and the second closest male differed by 1.7 ± 1.2 (mean ± SD).

Infants spent 43% of their time with their mother, 11% with their preferred male, and 7% with any other male (time spent within 5 m proximity). Every focal hour they spent a mean of 2:05 min in close proximity (within 1.5 m) or body contact with their preferred male (1.28 times/focal hour) compared to a mean of 27 s/focal hour or 0.27 times/focal hour with other males.

### Testing Predictions of the Mating Effort Hypothesis

**Fig. 2:**
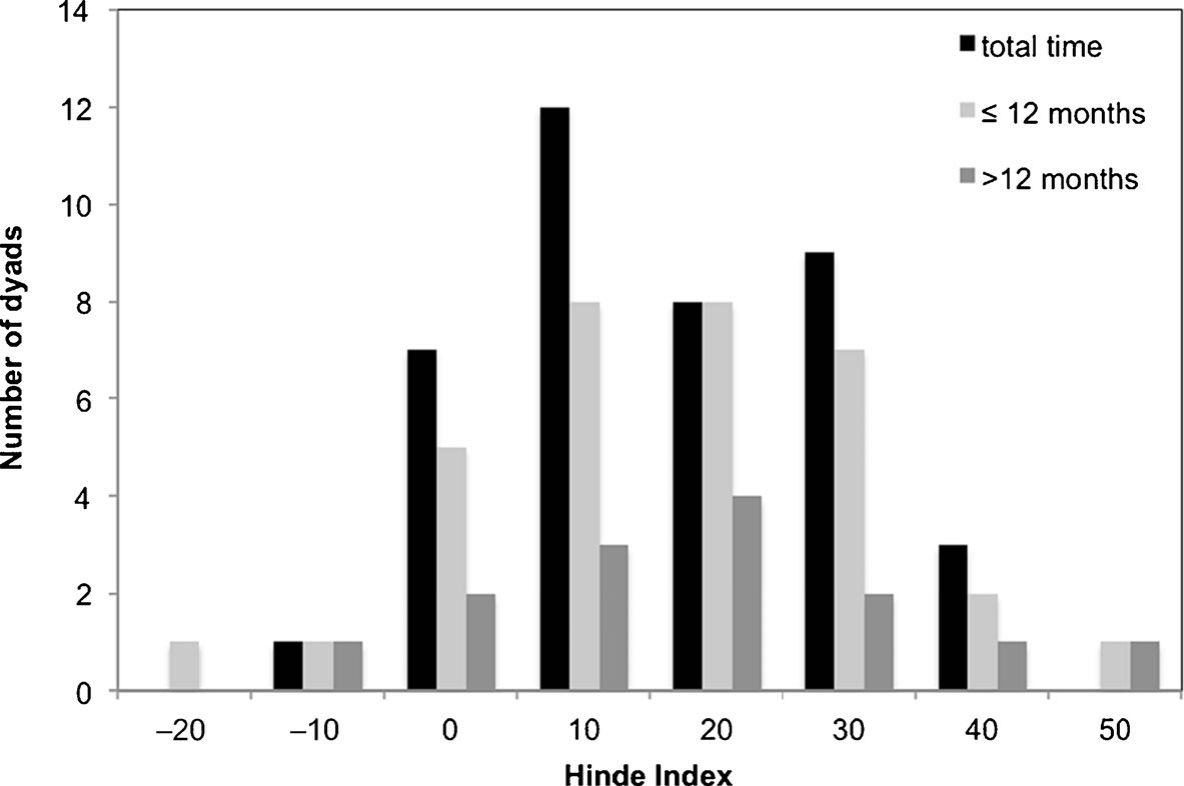
Histogram of Hinde indices as a measure of partners’ responsibility for maintaining immature–male association in a group of Assamese macaques at Phu Khieo Wildlife Sanctuary. Black = entire study period, light gray, before weaning (1–12 mo); dark gray = after weaning (13–21 mo). Positive values >10 indicate that immatures are responsible for initiating and maintaining close spatial proximity within 1.5 m (see Materials and Methods for details).

The Hinde indices of male–immature dyads were positive in 98% of cases (*N* = 40 male–immature dyads with >20 tolerated approaches) with a mean score ± SD of 20 ± 13, implying that immatures were responsible for maintaining close proximity to males much more than vice versa, which is in contrast to our prediction based on the mating effort hypothesis (Fig. 2). If split into the pre- (1–12 mo of age) and post- (13–21 mo of age) weaning period, 93% (*N* = 30 male–immature dyads with >20 tolerated approaches) of Hinde indices were positive with a mean score ± SD of 22 ± 14 before and 93% (*N* = 14 male–immature dyads with >20 tolerated approaches) positive indices postweaning with a mean ± SD of 22 ± 15 (Fig. 2). Immatures sought the proximity of their preferred male more than that of other males. The mean Hinde Index of all preferred male–immature dyads (mean ± SD = 29 ± 10) was 35% higher than the mean Hinde Index of all other male–immature dyads (mean ± SD = 14 ± 12, Wilcoxon signed rank test: *V* = 2, *Z* = –2.91, *N* = 12 (all focal infants), *P* = 0.001).

Immatures’ choice of the preferred male partner was stable. Across the entire study period and averaged across immatures, immatures chose the same preferred male partner from month to month in 80% of cases (Consistency Index mean ± SD = 0.79 ± 0.16, range: 0.38–0.94). All of the seven offspring of mothers that conceived again during the study period maintained their strong relationship with their preferred male partner on conception of their sibling, which again is in contrast with our prediction based on the mating effort hypothesis.

### Testing Predictions of the Paternal Care Hypothesis: Temporal Stability of Differentiation of Male–Immature Associations Across the First 21 mo of Life

**Fig. 3:**
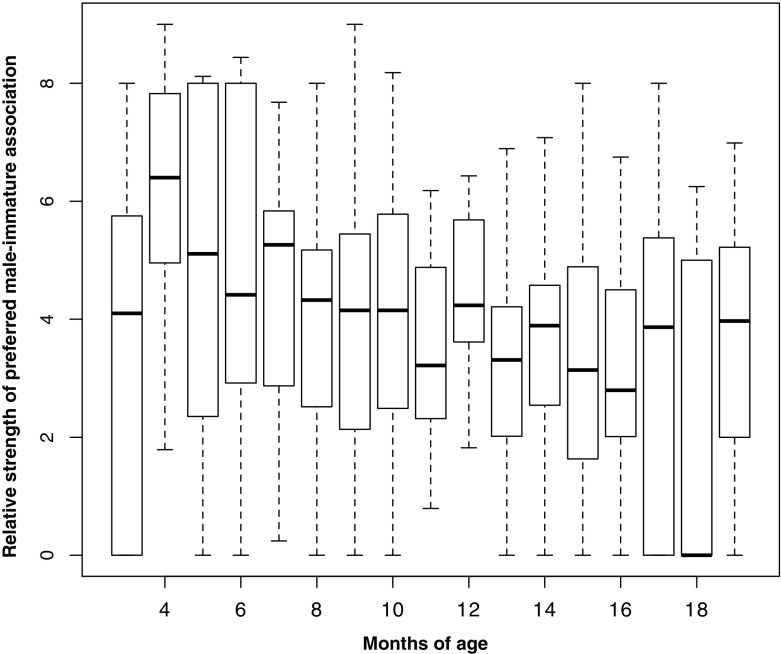
Temporal stability of the differentiation in immature–male associations from 3 to 19 mo of age in Assamese macaques at Phu Khieo Wildlife Sanctuary. Depicted are medians and 25%–75% percentiles across immatures of their Association indices with preferred males. Association indices are frequency and duration of close association within 1.5 m with the preferred male relative to the mean across males for a given immature.

Based on the Association Index the relative strength of the association with the preferred male exceeded the mean across males in 82% of immatures for every single month of life (mean ± SD = 10.5 ± 1.42 immatures per month; Fig. 3). This degree of preference or the strength of male–immature associations differed significantly for pre- (median= 4.31; interquartile range = 3.66–5.11) and postweaning periods (median = 3.43; interquartile range = 2.39–4.15; Wilcoxon signed rank test: *V* = 8, *Z* = –2.43, *N* = 12 (all focal infants), *P* = 0.01), a finding consistent with predictions based on the anti-infanticide hypothesis, but not between early (3– 6 mo; median = 4.30; interquartile range = 3.52 –5.67) and late infancy (7–12 mo; median = 3.92; interquartile range = 3.52–5.03; Wilcoxon signed rank test: *V* = 18; *Z* = –1.33, *N* = 11 (one infant excluded due to insufficient data during this period), *P* = 0.21).

**Fig. 4:**
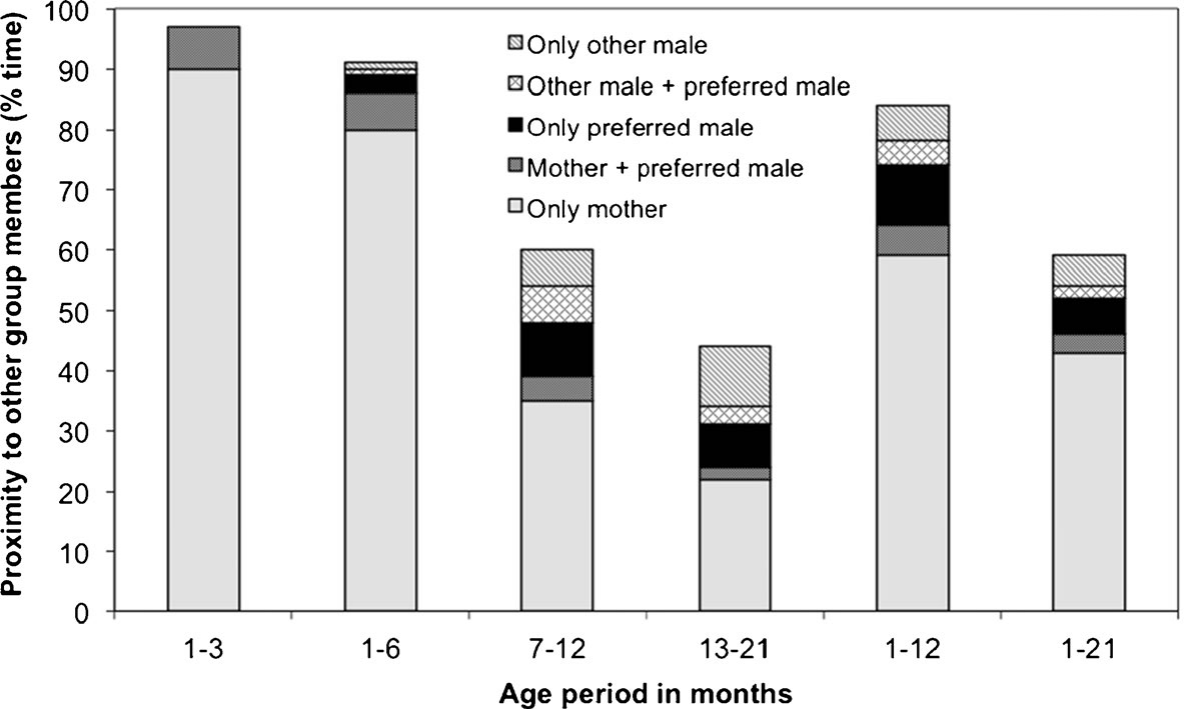
Time immature Assamese macaques from Phu Khieo Wildlife Sanctuary spent in proximity (≤5 m) to different group members for different immature age intervals expressed as proportion of active time.

In contrast to predictions from the anti-infanticide hypothesis and consistent with predictions from anti-harassment hypothesis, the absolute time that immatures spent in proximity to their preferred male did not differ between the pre- and postweaning phase (two-sided Wilcoxon signed rank test, *V* = 28, *Z* = –0.86, *N* = 12 (all focal infants), *P* = 0.42; Fig. 4). Time spent close to mothers significantly declined after weaning (two-sided Wilcoxon signed rank test, *V* = 0, *Z* = –3.06, *N* = 12 (all focal infants), *P* < 0.001). Time spent with group members other than preferred males or mothers doubled from the pre- to the postweaning phase (Fig. 4).

**Fig. 5:**
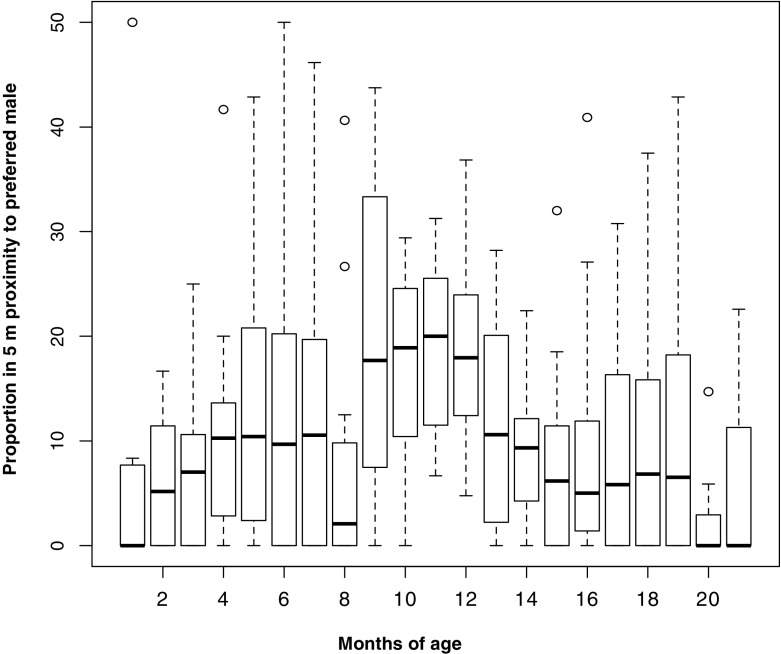
Time immature Assamese macaques from Phu Khieo Wildlife Sanctuary spent in proximity to preferred male across months of life expressed as proportion of active time. Depicted are medians and 25%–75% percentiles across immatures.

Comparing associations on smaller timescales revealed that until infants were 3 mo of age mothers always were in sight, when immatures spent time in proximity to the preferred male (7%) and nearly always until 6 mo of age (9% with the preferred male and of these 6% with mothers as well; Fig. 4). Once infants were 7 mo old they spent twice as much time in proximity to their preferred male (18% 7–12 mo of age vs. 9% 1–6 mo of age, two-sided Wilcoxon signed rank test *V* = 12, *Z* = –2.12, *N* = 12 (all focal infants), *P* = 0.03). This change was associated with a significant decrease in the time immatures spent with their mothers (Wilcoxon signed rank test, *V* = 0, *Z* = –3.06, *N* = 12, *P* = 0.001; Fig. 4). There was no difference in time spent in proximity to preferred males between late infancy (7–12 mo of age) and the postweaning phase (13–21 mo of age, two-sided Wilcoxon signed rank test, *V* = 18, *Z* = –1.65, *N* = 12, *P* = 0.11; Fig. 5).

The preferred male was more likely to be in proximity (≤ 5 m) in the presence of a second male, i.e., a potential aggressor, before weaning (*R*^2^ full model = 0.21, full vs. null-model χ^2^ = 39.35, *P* < 0.001), after weaning (*R*^2^ full model = 0.26, full vs. null-model χ^2^ = 26.73, *P* < 0.001) and across the entire study period (*R*^2^ full model = 0.18, full vs. null-model χ^2^ = 41.15, *P* < 0.001; Table III). The mother’s presence (potential protector) had a significant negative effect on the likelihood that the preferred male was in proximity across the study period. This effect was pronounced in the preweaning period compared to the post-weaning period (Table III). When mothers were absent (Fig. 4), immatures were associated with their preferred male for 24% of their time (10% with other males) during the first 12 mo of life compared to 11% (10% with other males) after weaning. Taken together, these results support predictions derived from the anti-harassment and from the anti-infanticide hypothesis.

**Table III:** Predictors of preferred male presence in proximity (≤5 m) of an immature Assamese macaque at Phu Khieo Wildlife Sanctuary

Age period	Fixed factors	Estimate ± SE	*z*-value	*P*
1–21 mo	Mother in sight (yes)^a^	–0.53 ± 0.14	–3.75	<0.001
	Other adult male in sight (yes)^a^	0.87 ± 0.14	6.03	<0.001
1–12 mo	Mother in sight (yes)^a^	–0.94 ± 0.18	–5.35	<0.001
	Other adult male in sight (yes)^a^	0.83 ± 0.19	4.45	<0.001
13–21 mo	Mother in sight (yes)^a^	–0.51 ± 0.20	–2.57	<0.01
	Other adult male in sight (yes)^a^	0.96 ± 0.19	5.00	<0.001

Results of three GLMMs controlling for immature identity and group composition with number of observations: 1–21 mo, *N* = 5526; 1–12 mo, *N* = 3240; 13–21 mo, *N* = 2286 proximity scans.

^a^Reference category: no.

### Influence of the Preferred Male on the Rate of Aggression Received/Submission Given by Immatures

Overall immatures received a mean ± SD of 0.75 ± 0.24 bouts of aggression per hour. Aggressors were either other immatures up to age 5 (60%) or adults (40%). The average rates of aggression received were significantly higher after weaning than before (Wilcoxon signed rank test, *V* = 1, *Z* = –2.98, *N* = 12 (all focal infants), *P* < 0.001), driven mainly by increased aggression received from other immatures. Aggression against mothers carrying immatures was not included in this calculation, which may partly explain the lower preweaning rates.

Although full models were different from null models, the presence of the preferred male in proximity (≤ 5 m) did not reduce the rate of aggression immatures received from conspecifics (*R*^2^ full model = 0.20, full vs. null-model χ^2^ = 5.32, *P* = 0.02) or reduce the rate of submission given to conspecifics across the study period (*R*^2^ full model = 0.16, full vs. null-model χ^2^ = 5.68, *P* = 0.017; Table IV). The rate of aggression received and submission given, in fact, significantly increased in the presence of the preferred male. This was probably due to the mild aggression given by preferred males that spent more time with the infant in proximity, which is not controlled for in this analysis. After excluding the mild aggression given by preferred males to immatures, the resulting full model of aggression received did not differ from the null model with only the random factors and the intercept (full vs. null-model χ^2^ = 0.02, *P* = 0.89). Likewise, after excluding the submission given by immatures to their preferred male, the presence of the preferred male no longer predicted the rate of given submissions toward group members (full vs. null-model χ^2^ = 0.21, *P* = 0.65). These results do not support our predictions based on the anti-harassment hypothesis.

**Table IV:** Influence of preferred male (PM) presence on the likelihood of immatures receiving aggression (including aggression received by PM) and giving submission (including submission given to PM) in Assamese macaques at Phu Khieo Wildlife Sanctuary

Response variable	Fixed factors	Estimate ± SE	*z*-value	*P*
Received aggression (incl. PM)	Pref. males in sight (yes)^a^	0.57 ± 0.25	2.27	0.023
Given submission (incl. PM)	Pref. male in sight (yes)^a^	0.48 ± 0.20	2.37	0.018

GLMMs controlling for immature identity (*N* = 12) and immature sex and local group composition (*N* = 167). Number of observations: 1774. SE = standard error.

^a^Reference category: no.

### Agonistic Support for Immatures

**Fig. 6:**
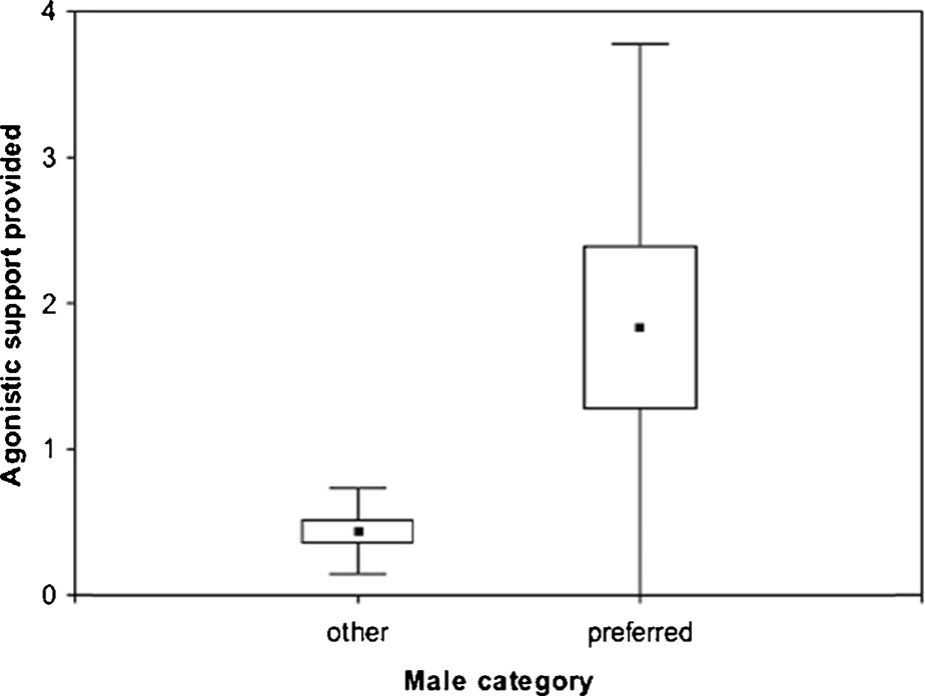
Agonistic support provided by preferred and the average other male to infant Assamese macaques at Phu Khieo Wildlife Sanctuary during the study period May 2011 to December 2012. Values are averages across immatures (midpoint), standard error (boxes), and standard deviation (whiskers).

In line with the anti-harassment hypothesis, adult males intervened on behalf of immatures in 50% of adult interventions. For 75% of immatures the preferred male provided more support than any other male. Immatures received support more than four times as often from their preferred male (mean ± SD = 1.8 ± 1.9) than from the average nonpreferred male (mean ± SD = 0.4 ± 0.3; Wilcoxon signed rank test: *V* = 10.5; *Z* = –2.24, *N* = 12 (all focal infants), *P* = 0.02), a difference that is exacerbated if support by close runner-ups to the preferred male are included for six immatures (preferred and runner-ups: mean ± SD = 1.7 ± 1.4 vs. nonpreferred males: mean ± SD = 0.3 ± 0.2; Fig. 6). Treating immature preference as a continuous variable, the rate of agonistic support received from a male was significantly predicted by the strength of the affiliative relationships between the immature and the male; the higher the CSI between the immature and the male the more often he supported the immature (*R*^2^ full model = 0.35, full vs. null-model χ^2^ = 28.58, *P* < 0.001, estimated *z*CSI = 0.58 ± 0.002, *P* < 0.001).

## Discussion

Based on our relatively small sample of immatures from a single group from birth through their infancy into their early juvenile period, we conclude that Assamese macaque immatures and males form highly differentiated affiliative relationships. All but one immature had one or two relationships that was stronger than the average male–immature relationship in the group and was clearly set apart in strength and immature responsibility from relationships an immature had with other males. In the following we discuss whether such nonrandom patterns of affiliative relationships would evolve as male mating effort or male parental effort and which form of parental care may be provided to the immature.

Our predictions derived from the mating effort hypothesis about immature–male relationships were not met. Immatures, not males, were responsible for maintaining close spatial proximity. That immatures approach males more often than vice versa seems to contrast Assamese macaques from rhesus macaques, in which social interactions are initiated more often by males than immatures (Langos *et al*. 2013). The infant’s greater initiative in relationship maintenance compared to the male may be mediated by the mother as a means to foster male–infant association (Widdig 2007). Both the strength of the relationship and partner choice for the preferred male were stable through the pre- and well into the postweaning period. Following the rationale of the mating effort hypothesis, if males affiliated with immatures only to endear themselves to the mothers in the expectation of mating privileges, males should abandon immatures on the conception of the subsequent offspring, as mating privileges will not lead to increased reproductive success from this point on. Even if males are unaware of the timing of conception as in Assamese macaques, they seem to be aware of whether females will conceive during a given mating season (Fürtbauer *et al*. 2011a). Relationships of immatures with their preferred partners were not affected by their mother conceiving again. Thus, the classical mating effort hypothesis alone cannot explain why immatures establish special relationships with certain males.

We have previously shown for the same group using spatial nondirectional data that the time a male spends in proximity to an immature is predicted both by paternity and by the time the male spent with the mother around the time the immature was sired (Ostner *et al*. 2013). The results of the present study indicate that the male–immature association is established and maintained by the immature and its mother rather than the male. The main association partners of immature Assamese macaques are often their sires or males with high estimates of paternity certainty, as in chacma baboons and rhesus macaques (Huchard *et al*. 2010; Langos *et al*. 2013; Moscovice *et al*. 2009, 2010) but not in yellow baboons (Nguyen *et al*. 2009). Therefore, any help given by males to immatures may be interpreted as paternal care (*cf*. Alberts and Fitzpatrick 2012).

This study did not provide clear evidence in support of the anti-infanticide hypothesis of paternal care. The degree of differentiation in immature–male relationships did not follow gradual changes in the risk of infanticide with immature age but decreased when infanticide risk ceased after weaning. The absolute time the preferred male spent around the immature increased instead of decreased after 6 mo of age, i.e., after the period of maximum infanticide risk, and then remained stable until the end of our study period. Paternity confusion may be so effective in Assamese macaques (Fürtbauer *et al*. 2011a) that infanticide from within the group is rare and possibly maladaptive. In this study the risk of infanticide by recent immigrants was zero because no adult males immigrated during the study period or the preceding 6 mo. In rhesus macaques only genetic sires, but not nonsires, affiliate more with immatures during infancy, i.e., when vulnerability is high, compared to later juvenility (Langos *et al*. 2013). Within the baboon clade chacma baboons are characterized by a higher incidence of infanticide compared to olive and yellow baboons (Palombit 2003), and consequently, male–mother–immature associations in olive and yellow baboons may provide females with protection against nonlethal aggression or promote future male–juvenile bonding rather than being explained by infanticide avoidance (Lemasson *et al*. 2008; Nguyen *et al*. 2009). Apart from variation in infanticide risk, variation may exist in the effectiveness of protection by the mother. It has been argued that females may be effective protectors against male infanticide in species with smaller sexual dimorphism in body size and weaponry (Palombit 2003), which may apply to Assamese macaques. Thus, male protection from male infanticide either is so effective that it is rarely observed (van Schaik 2000a) or male protection is irrelevant either because immigration rates are low and paternity certainty within groups is distributed or because females are effective protectors.

Predictions derived from the anti-harassment hypothesis of paternal care were partly met. The time immatures spent in proximity to preferred males was relatively constant throughout the observation period and did not drop after weaning. The presence of the preferred male in proximity of an immature, however, did not reduce the probability of receiving aggression from any group member, because the preferred males themselves sometimes acted aggressively against the immatures. Even after excluding interactions between the immature and its preferred male we did not find support for reduced aggression received, submission given, or aggression given by immatures, which is in contrast to chimpanzee males that are less aggressive toward their genetic offspring compared to unrelated immatures (Lehmann *et al*. 2006). The benefit may come in the form of protection against others: Yellow baboon infants in association with those males that spent most time with their mother receive less harassment from other females and generally utter fewer distress calls (Nguyen *et al*. 2009), but these males are often not their sires or likely sires. In our detailed situational analyses preferred males were present more often when the mother as alternative protector was absent and when other males as potential aggressors were present. The latter results mimic findings for chacma baboons (Huchard *et al*. 2013). Most crucially, in the present study preferred males acted as protectors, because they supported the immature four to five times more often than the average male as described for chacma and yellow baboons (Moscovice *et al*. 2009).

Follow-up work to our study should focus on the opportunity to test conclusively whether males preferentially support their own over others’ immature offspring (Buchan *et al*. 2003) and long-term data to test whether the presence of sires affects offspring fitness (Charpentier *et al*. 2008). It has been hypothesized that this true paternal care is more common in species exhibiting exaggerated sexual swellings as reliable indicators of ovulation probability, where males have high paternity certainty selecting for differential investment in genetic offspring (Alberts and Fitzpatrick 2012). Like all macaques of the sinica group clade (Maestripieri 1998), Assamese macaques show frequent male–immature association and affiliation but lack reliable indicators of ovulation (Fürtbauer *et al*. 2010). Yet, in our study population genetic paternity is predictive of male–immature association also after weaning when mother–immature association is reduced (Ostner *et al*. 2013), and results of the present study indicate that this association is maintained by the immature, is risk sensitive, and benefits the immature in terms of enhanced agonistic support. Thus, true paternal care may evolve in the absence of good ovulation indicators. Barbary and rhesus macaque females provide better cues to ovulation than Assamese macaques but less precise ones than yellow and chacma baboons (Brauch *et al*. 2007; Dubuc *et al*. 2009; Pfefferle *et al*. 2011; Young *et al*. 2013). In rhesus macaques male-immature associations, nevertheless, are predicted by paternity (Langos *et al*. 2013) and are associated with faster growth (Langos *et al*. 2015). Unlike in yellow baboons, coresidence with the sire did not, however, affect the offspring’s lifetime reproductive success in rhesus macaques (Langos *et al*. 2015). In Barbary macaques male–immature association is not predicted by paternity (Ménard *et al*. 2001; van Schaik and Paul 1996) and may be selected for the benefits it confers to males in agonistic buffering and male bonding (Berghänel *et al*. 2011; Paul *et al*. 1996), whereas short- and long-term benefits for immatures remain unknown. The crucial data for a comparative test of the hypothesis that true paternal care evolved together with precise indicators of ovulation are not yet available. We hope this study stimulates further research, as the results suggest a complex picture.
